# Job Satisfaction Among Graduate Medical Education Trainees

**DOI:** 10.7759/cureus.75258

**Published:** 2024-12-07

**Authors:** George W Koutsouras, Jamie Romeiser, Danielle A Katz

**Affiliations:** 1 Neurosurgery, Upstate University Hospital, Syracuse, USA; 2 Public Health and Preventive Medicine, State University of New York Upstate Medical University, Syracuse, USA; 3 Graduate Medical Education, Upstate University Hospital, Syracuse, USA

**Keywords:** burnout, graduate medical education, job satisfaction, primary survey, survey, wellness

## Abstract

Burnout among medical residents and fellows (postgraduate trainees) has been receiving significant attention in the scientific literature with far less focus on the factors that correlate with job satisfaction and well-being. A better understanding of the characteristics that increase job satisfaction (rather than just those that lead to burnout) may allow programs to develop and enhance those positive features, conceivably leading to improved mental health, retention, and recruitment. We hypothesize that job satisfaction among postgraduate trainees is positively impacted by feeling that their work is meaningful, that their work schedules are equitable, and that they are appreciated by their faculty. A cross-sectional 28-item questionnaire was sent to 613 residents and fellows in a single institution to measure multiple items with relevance to job satisfaction, happiness, well-being, meaning, and burnout. Correlative, bivariate, and multivariable regression analyses were used to assess for factors associated with these elements, and a composite summary score of them. Feeling appreciated by faculty and satisfaction with time outside of work also were associated with different elements of job satisfaction and well-being. Burnout was negatively associated with satisfaction, and, in a composite model, burnout was significantly moderated by satisfaction with opportunities for growth. The authors identified factors that correlate with job satisfaction among a representative sample of postgraduate trainees at an academic institution. These elements may be used to guide efforts to support job satisfaction and the well-being of residents and fellows.

## Introduction

Burnout among physicians, including residents and fellows (postgraduate trainees), has received significant attention in the scientific literature [1-8], but far less has been described about the factors that correlate with job satisfaction and overall well-being [2,9-13]. We believe that the results of this study can be utilized to develop strategies to increase resident and fellow job satisfaction and well-being.

Finding meaning in one’s work has been identified as a component of well-being and a negative predictor of burnout among various healthcare workers [14]. Focusing more specifically on residents, burnout has been shown to negatively correlate with having enough time to fulfill both professional and personal responsibilities, having some control/voice regarding training programs/schedules, and receiving recognition of accomplishments [10]. Some suggest that programs and institutions could help prevent burnout in residents by cultivating the components of the experience that allow and encourage residents to find meaning in their work [2].

We hypothesize that job satisfaction among postgraduate trainees is positively impacted by feeling that there is meaning in their work, that they are appreciated by those with whom they work, and that their work schedules are equitable.

This study aims to investigate these factors by examining correlations between job satisfaction and variables such as burnout, faculty support, work schedule equity, and mentorship. Specifically, we seek to quantify the impact of these factors on overall trainee well-being and develop recommendations for enhancing trainee satisfaction.

## Materials and methods

Our institution is an academic medical center with 53 residency and fellowship programs. The institution’s overall postgraduate trainee population at the time of survey distribution was 83.0% residents and 17.0% fellows. We conducted a single-institution cross-sectional study using a self-report survey developed by the authors and distributed through REDCap (Research Electronic Data Capture) software in 2023 (for the survey, see Appendix Figures 3-5).

We were unable to identify a previously validated survey that measured the specific areas in which we were interested as it pertains to factors influencing job satisfaction among residents and fellows. Therefore, we reviewed the literature for studies evaluating physician burnout and job satisfaction and used the questions in those studies to guide the development of our survey [3,9,13,15,16-20]. Questions were formulated over various domains, including questions in the areas of meaning, trust, equity, salary, personal time, and overall satisfaction. Likert scales were weighed from 1 to 6, with responses ranging from very negative feelings to very positive feelings. Though burnout may have a bidirectional relationship with job satisfaction within medical professions, a recent longitudinal study of medical students suggests that burnout may unidirectionally and negatively predict satisfaction [6,8]. Therefore, we used a validated single-item question to measure the level of burnout [4].

Questions on basic demographic information included position (resident or fellow), postgraduate year in training (PGY 1, 2, 3, 4+), specialty (medicine, including emergency medicine and fellowships, family medicine, internal medicine and subspecialty fellowships, neurology and subspecialty fellowships, pediatrics and subspecialty fellowships, physical medicine and rehabilitation); surgery, including neurosurgery, orthopedic surgery, otolaryngology, general surgery, vascular surgery, urology; other (anesthesiology, psychiatry, obstetrics and gynecology, ophthalmology, pathology and subspecialty fellowships, radiology and subspecialty fellowships, radiation oncology)), gender (male, female, non-binary), hours worked per week (≤40, 41-50, 51-60, 61-70, 71-80, >80), and presence of a mentor (Yes/No).

We then conducted a pilot study in which the survey was sent to approximately 10% of the institutional postgraduate trainees, across specialties and postgraduate year (PGY) levels. Qualitative feedback was obtained on the clarity of the questions. Time to survey completion, which was captured as an automated feature of the REDCap survey, was reasonable and ranged from two to seven minutes. Completion rates for each question were high. An initial correlation matrix for the Likert scale questions demonstrated numerically positive correlations between satisfaction and most questions and a negative correlation for burnout, demonstrating initial face validity. After minor rephrasing revisions, the final survey was sent to all university residents and fellows not included in the pilot study group (N = 613). We made several efforts to maximize response rates, including keeping the survey brief to minimize the burden of time, and pre-testing the invitations through the pilot study. Further, one initial request and three reminders were sent over the course of four weeks. These reminders were sent on various days of the week/times of the day to potentially reach a broader audience and maximize participation. Additionally, the survey was completed anonymously to further encourage participation and reduce non-response bias. 

Individual surveys were excluded if over 50% of the survey was left incomplete. If greater than 50% of the survey was completed, then the completed items were used in the analysis. Descriptive and frequency statistics are presented in Table 2. Correlations between the survey questions were described using Spearman’s correlation matrix. Bivariate ordinal logistic regressions were performed to examine associations with the primary outcome, which was overall satisfaction with the residency/fellowship program. Ordinal regression was chosen due to the ordinal nature of the outcome. Additionally, a multivariable ordinal stepwise regression was used to determine the most important factors associated with overall satisfaction while reducing issues of multicollinearity. Proportional odds assumptions were examined in the statistical output and satisfied for all models.

During analysis of the data, the five survey questions that assessed overall satisfaction, happiness, meaning, well-being, and enjoyment were found to be very highly correlated (Cronbach’s alpha = 0.93), and all variables had a high independent correlation with overall satisfaction (all spearman’s rho > 0.64). Both findings indicated that these questions may be measuring the same construct. Therefore, two analytical approaches were taken to further assess what role other factors from the survey might have in determining overall satisfaction. First, a multivariable ordinal stepwise logistic regression for satisfaction was conducted without the questions of happiness, meaning, well-being, and enjoyment. Second, a composite outcome variable was constructed by summing responses for overall satisfaction, happiness, meaning, well-being, and enjoyment, and a multiple linear regression model was trained based on stepwise regression. All variance inflation factor estimates were less than 3. All analyses were performed using SAS 9.4 © (Cary, NC) at the α = 0.05 significance level. This study was granted exempt status by the Institutional Review Board (IRB). Data are available upon special request and approval from the authors.

## Results

Out of the 613 surveys sent, there were 207 survey responses. Of these, two were removed for having over 50% of the responses missing, for a final sample size of 205 (33% response rate). The demographics of the respondents largely reflected the overall composition of the postgraduate trainees at the institution (Table 1). Most (165/205) respondents were residents (80.5%), with 38/205 (18.5%) identifying as fellows and 2/205 (1%) not responding. When broken down further by PGY in training, the overall distribution of postgraduate trainees within the institution at that time was 23.6% PGY-1, 21.6% PGY-2, 21.8% PGY-3, and 33.0% PGY-4 or higher. The survey respondents followed a similar distribution with PGY-1 and 2 years being slightly under-represented and PGY-3 and higher being slightly over-represented. In the survey question, there was not a descriptor as to which specialty areas were specifically to be considered as “Medical”, “Surgical” and “Other”, but there did not appear to be one type of specialty area that was significantly over- or under-represented. Finally, the gender identification of the survey respondents was largely consistent with institutional data regarding the gender of trainees (institutionally 59.4% male, 40.4% female, 0.1% non-binary/prefer not to answer), with the exception that a significantly higher number identified as non-binary/prefer not to answer on the survey, and perhaps a slight under-representation of males among survey respondents.

**Table 1:** Demographics

Characteristics	n	%
Total	205	100%
Position		
Resident	165	80.5%
Fellow	38	18.5%
No Response	2	1.0%
Years Training		
PGY-1	41	20.0%
PGY-2	33	16.1%
PGY-3	50	24.4%
PGY-4+	77	37.6%
No Response	4	2.0%
Hours Worked/Week		
<=40	9	4.4%
41-50	43	21.0%
51-60	50	24.4%
61-70	39	19.0%
71-80	50	24.4%
>80	12	5.9%
No Response	2	1.0%
Specialty		
Medical	110	53.7%
Surgical	63	30.7%
Other	31	15.1%
No Response	1	0.5%
Gender		
Male	107	52.2%
Female	86	42.0%
Prefer not to respond	12	5.9%
Mentor		
Yes	158	77.1%
No	47	22.9%
Level of Burnout		
No Symptoms	18	8.8%
Occasional symptoms	99	48.3%
Experiencing 1+symptoms	66	32.2%
Symptoms won't go away	14	6.8%
Complete Burnout	6	2.9%
No Response	2	1.0%

**Figure 1:**
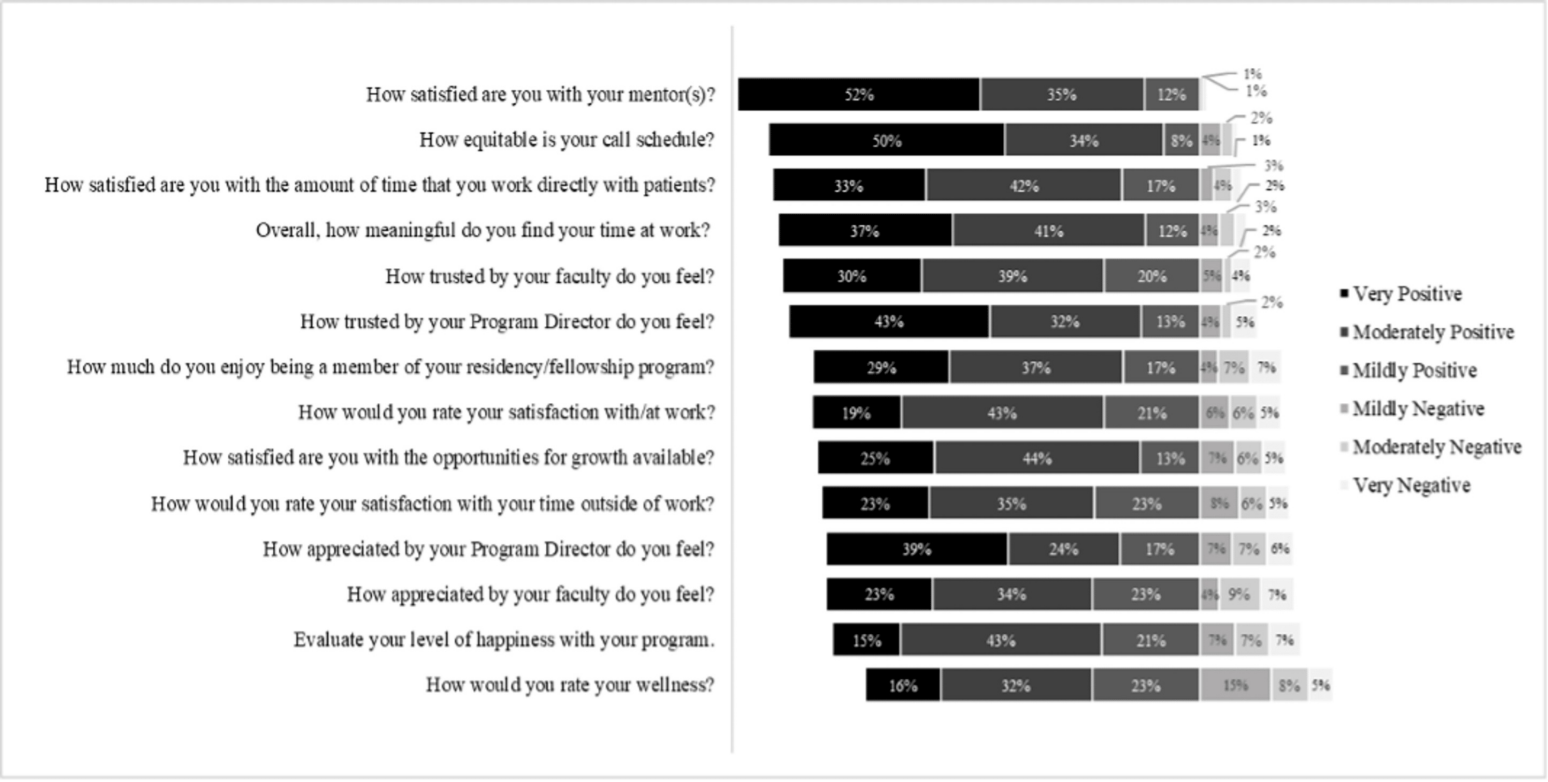
Cumulative Distribution of Survey Responses by the Highest Distribution of Positive Responses

Likert scale questions and responses are displayed in Figure 1. Results are displayed by the questions with the largest proportions of positive responses at the top. Mean response scores are displayed in Table 1. A total of 39/205 (19%) respondents reported being very satisfied with/at work, 88/205 (43%) were moderately satisfied, 41/205 (21%) were mildly satisfied, 13/205 (6%) were mildly dissatisfied, 12/205 (6%) were moderately dissatisfied, and 10/205 (5%) were very dissatisfied. The average overall satisfaction score was 4.49 (SD = 1.31, median = 5 (IQR = 4, 5)). Questions with the highest proportion of very positive responses included questions on satisfaction with mentorship (of the 158 with a mentor, 82 responses (52%)), call schedule equity (103/205 responses (50%)), and feeling both trusted and appreciated by the program director (88/205 (43%) and 80/205 (39%), respectively). This differed somewhat from the question with the highest average scores, which included satisfaction with mentorship (mean = 5.37, SD = 0.77), equity of call schedules (mean = 5.23, SD = 1.04), and feeling trusted by the program director (mean = 4.93, SD = 1.35), but also included time spent directly with patients (mean = 4.91, SD = 1.13), meaning in the time spent at work (mean = 4.98, SD = 1.16), and feeling trusted by the faculty (mean = 4.78, SD = 1.21) (Table 2).

**Table 2:** Bivariate Ordinal Logistic Regression Predicting Satisfaction

Predicting Overall Satisfaction with Program	Bivariate Ordinal Regression	
	Odds Ratio (95% CI)	P-value
Enjoyment	5.47 (4, 7.46)	<0.001
Satisfaction with opportunities	4.77 (3.55, 6.41)	<0.001
Time at work is meaningful	5.49 (3.9, 7.72)	<0.001
Happiness	7.75 (5.42, 11.08)	<0.001
Satisfied with amount of time with patients	2.96 (2.27, 3.88)	<0.001
Appreciated by the program director	2.22 (1.83, 2.71)	<0.001
Appreciated by faculty	3.57 (2.78, 4.58)	<0.001
Trusted by the program director	2.01 (1.64, 2.48)	<0.001
Trusted by faculty	3.61 (2.74, 4.76)	<0.001
Identified a mentor (yes/no)	3.33 (1.81, 6.1)	<0.001
Satisfaction with mentor	2.9 (1.92, 4.37)	<0.001
Equitable call service	2.37 (1.82, 3.09)	<0.001
Satisfaction with time outside of work	2.57 (2.05, 3.22)	<0.001
Wellness	3.96 (3.04, 5.15)	<0.001
Burnout	0.17 (0.12, 0.25)	<0.001
Years in training	1.04 (0.83, 1.29)	0.76
Hours per week at work	0.61 (0.5, 0.75)	<0.001
Gender (female vs. male)	1.12 (0.66, 1.88)	0.68
Specialty (medical vs. surgical)	1.97 (1.11, 3.49)	0.01
Specialty (other vs. surgical)	0.93 (0.43, 2.01)	0.24

With the exception of gender and years in training, all questions were significantly associated with overall satisfaction in the bivariate models (Table 2). The odds of high satisfaction increased with increasing levels of positive responses. For example, as participants reported increasing levels of feeling appreciated by the faculty, the odds of high satisfaction significantly increased (OR = 3.57 (CI = 2.78, 4.58)). However, as levels of burnout increased, the odds of high satisfaction significantly decreased (OR = 0.17, (CI = 0.12, 0.25)).

All factors from Table 3 were entered into a stepwise regression model, which identified four primary factors associated with overall satisfaction: overall happiness, meaningful time at work, overall well-being, and feeling appreciated by the faculty (Model 1). Because happiness, well-being, meaning, and enjoyment were strongly correlated with overall satisfaction and were thought to be measuring similar constructs, the same modeling process was repeated but with a limited model that excluded these factors (Model 2). Separate exploratory stepwise models were also conducted to predict happiness (Model 3), well-being (Model 4), and meaning (Model 5), with the limited variable listing. An exploratory model was also examined for enjoyment, but the proportional odds assumption was not met and therefore model results are not reported.

**Table 3:** Stepwise Ordinal Regression for Overall Satisfaction, Happiness, Meaning, and Wellness Models 2, 3, 4, and 5 were trained without the inclusion of overall satisfaction, happiness, wellness, meaning, and enjoyment.

	Overall Satisfaction (Full, Model 1)			Overall Satisfaction (Model 2)			Happiness (Model 3)			Wellness (Model 4)			Meaning (Model 5)		
	OR	95% CI	P	OR	95% CI	P	OR	95% CI	P	OR	95% CI	P	OR	95% CI	P
Happiness	3.50	(2.33, 5.25)	<0.001	-	-	-	-	-	-	-	-	-	-	-	-
Wellness	1.66	(1.2, 2.31)	0.002	-	-	-	-	-	-	-	-	-	-	-	-
Time at work is meaningful	2.20	(1.49, 3.25)	<0.001	-	-	-	-	-	-	-	-	-	-	-	-
Appreciated by faculty	1.61	(1.21, 2.15)	0.001	1.92	(1.42, 2.59)	<0.001	1.71	(1.28, 2.27)	<0.001	-	-	-	-	-	-
Satisfaction with opportunities				2.35	(1.67, 3.32)	<0.001	3.15	(2.2, 4.52)	<0.001	1.66	(1.26, 2.18)	<0.001	1.82	(1.34, 2.48)	<0.001
Satisfied with amount of time with patients				1.85	(1.36, 2.52)	<0.001	1.62	(1.2, 2.2)	0.002	1.45	(1.06, 1.97)	0.02	2.39	(1.75, 3.27)	<0.001
Burnout				0.44	(0.28, 0.69)	<0.001	0.60	(0.38, 0.92)	0.02	0.13	(0.08, 0.22)	<0.001	-	-	-
Satisfaction with time outside of work										2.84	(2.11, 3.81)	<0.001	1.31	(1.07, 1.75)	0.01
Trusted by faculty													1.73	(1.27, 2.36)	0.001
c-Index	0.91			0.88			0.86			0.89			0.84		

Interestingly, satisfaction with opportunities for growth and satisfaction with the amount of time spent with patients was significantly correlated with satisfaction, happiness, well-being, and meaning. Feeling appreciated by faculty was positively associated with satisfaction and happiness. Satisfaction with time outside of work was positively associated with well-being and meaning. Feeling trusted by faculty was positively associated with meaning. Burnout was negatively associated with satisfaction, happiness, and well-being. In the stepwise linear regression model for the composite outcome, satisfaction with opportunities, satisfaction with the amount of time spent with patients, feeling appreciated by faculty, satisfaction with time outside of work, and burnout were all significant factors associated with the satisfaction composite score (Table 4).

**Table 4:** Stepwise Linear Regression Model for the Composite Outcome

Parameter	Estimate	Pr > |t|
			Intercept	11.88	<0.0001
Satisfaction with opportunities	1.06	0.007
Burnout	-2.88	<0.0001
Interaction (satisf. opportunities and burnout)	0.31	0.01
Satisfaction with time outside of work	0.64	0.0002
Appreciated by faculty	0.69	0.0002
Satisfied with amount of time with patients	0.75	<0.0001

**Figure 2:**
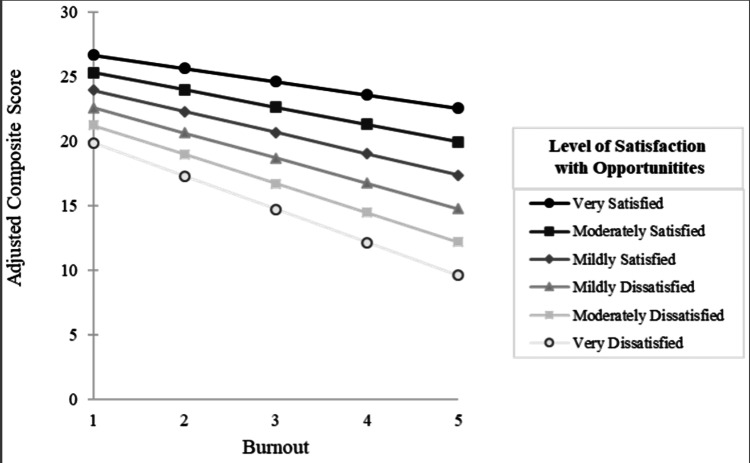
Association Between Burnout and Adjusted Composite Scores Line graph demonstrating the moderating effect of satisfaction with opportunities on the association between burnout and composite scores. As the levels of satisfaction with opportunities increased, the association (slope) between burnout and the composite scores became less negative.

There was also significant interaction between burnout and satisfaction with opportunities. Specifically, as levels of satisfaction with opportunities increased, the association (slope) between burnout and the composite score became less negative (Figure 2).

## Discussion

We present the results of a cross-sectional large institutional analysis, which demonstrates that job satisfaction among residents and fellows is strongly correlated with satisfaction with the opportunities for growth within their program and the satisfaction they have from the amount of time spent with patients. Four factors bivariately associated with overall job satisfaction were overall happiness, meaningful time spent at work, equitable work schedules, and feeling appreciated by faculty.

While overall happiness and overall well-being likely both impact job satisfaction and are impacted by job satisfaction, they are broader metrics for which it may be difficult for residency and fellowship programs to devise programs or strategies to specifically address. Training programs and institutions may be more able to have a direct effect on the meaningfulness of the time that residents and fellows spend at work and in promoting a feeling among residents and fellows that they are appreciated by the faculty [3,13,19]. Additionally, we identified a strong correlation between satisfaction with opportunities for growth on increasing job satisfaction and decreasing burnout. This element can be viewed as a specific and modifiable target. Trainees often aspire to be leaders and influencers within their institution, community, and their particular field. Cultivating opportunities for growth and development in these areas by welcoming trainees into hospital-wide committees, boosting research productivity, and creating a trainee-centric approach to fostering professional growth may enhance their job satisfaction and moderate the development and impact of burnout [7,20,21].

Overall job satisfaction, happiness with their program, meaningful time at work, and enjoyment in being a member of their program were all strongly correlated and therefore possibly assessing the same underlying construct. Therefore, we excluded these factors to try to assess the impact of other factors that were more distinct. The other factors that had a strong positive correlation with overall job satisfaction were satisfaction with opportunities for growth, feeling appreciated by faculty, and satisfaction with the amount of time spent with patients. Similarly, we identified factors that strongly correlated with overall well-being, happiness, and feeling that the time at work was meaningful. These included the amount of time spent with patients, satisfaction with the opportunities for growth in the program, feeling trusted by faculty, and satisfaction with time outside of work. The first three of these are domains that may be influenced by programs and faculty.

Burnout was negatively correlated with overall well-being and satisfaction but again moderated by satisfaction with opportunities for growth. Assessing well-being and burnout, and identifying those not satisfied with their job, has both professional and personal implications [22,23].

Training programs and institutions can have a direct effect on the meaningfulness of the time that residents and fellows spend at work and with patients, as well as promote efforts to have faculty show appreciation for the residents and fellows. Although there have been limits placed on duty hours since 2003, the evidence does not support that these restrictions alone reduce burnout rates [19]. Among a large cohort of other healthcare professionals, a strong driver of career satisfaction was observed to be the meaning they found in work [3]. In surgical fields, enhancing surgical experience, independence, and mentorship also are drivers of satisfaction [13]. It is quite possible that this approach of focusing on and fostering the positive may help curb the prevalence of burnout that is seen and discussed so often.

While the issues of postgraduate trainee job satisfaction, well-being, and burnout clearly are complex and interrelated, there appear to be opportunities to proactively create or modify an environment that supports resident and fellow job satisfaction by focusing on opportunities for professional growth, by encouraging faculty to let postgraduate trainees know that they are appreciated, by optimizing time spent with patients, and by assuring equity among schedules. In addition to identifying critical factors influencing trainee job satisfaction, this study’s strengths include a diverse sample of postgraduate medical trainees and the use of a composite satisfaction score, which offers a holistic view of job satisfaction dynamics. However, several limitations must be noted. The reliance on self-reported data may introduce bias, and as a single-institution study, findings may not be universally applicable. There are inherent limitations to this study as self-reported surveys are subject to selection bias and the possibility of differences in the interpretation of survey questions. We aimed to thwart any unreliability and inconsistencies in the survey by performing a pilot study (n = 62) prior to the administration of the final version of the survey. Our 33% response rate (n = 205), which is a satisfactory response rate from a global perspective, seems to provide a generalizable viewpoint as our cohort was similar in postgraduate year, gender, and specialty in comparison to the entire institutional population of postgraduate trainees, However, with a 33% response rate, it is important to consider the potential differences between respondents and non-respondents, and a higher response rate would have added greater validity to the interpretation of the results. Another inherent limitation is the single-institution cohort, which may limit generalizability across different types of institutions and geographic regions. Multi-institutional studies could validate these findings, focusing on similar factors in different contexts or regions.

Future directions may include the development of interventions based on these data (e.g. mentorship programs, work schedule adjustments, strategies to ensure enough time with patients, and increasing opportunities for growth) and then testing the impact on trainee job satisfaction and wellness.

## Conclusions

We provide a characterization of job satisfaction among a cross-sectional sample of postgraduate trainees at a single academic institution. Future studies may include administering this survey at other institutions to better assess the generalizability of these data to other institutions and across multiple institutions. Additionally, it may be useful to implement programs to encourage the contributors to job satisfaction (time with patients, finding meaning in work, appreciation shown by faculty, equity of schedules, opportunities for growth within the program) and to assess the impact that they have on postgraduate trainee satisfaction and well-being.

## Figures and Tables

**Figure 3:**
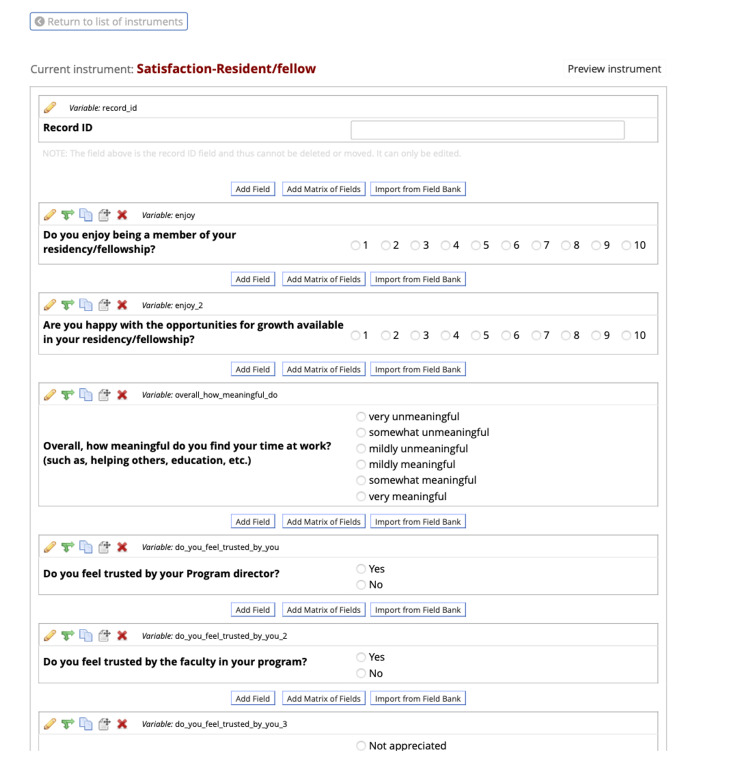
Survey Page 1

**Figure 4:**
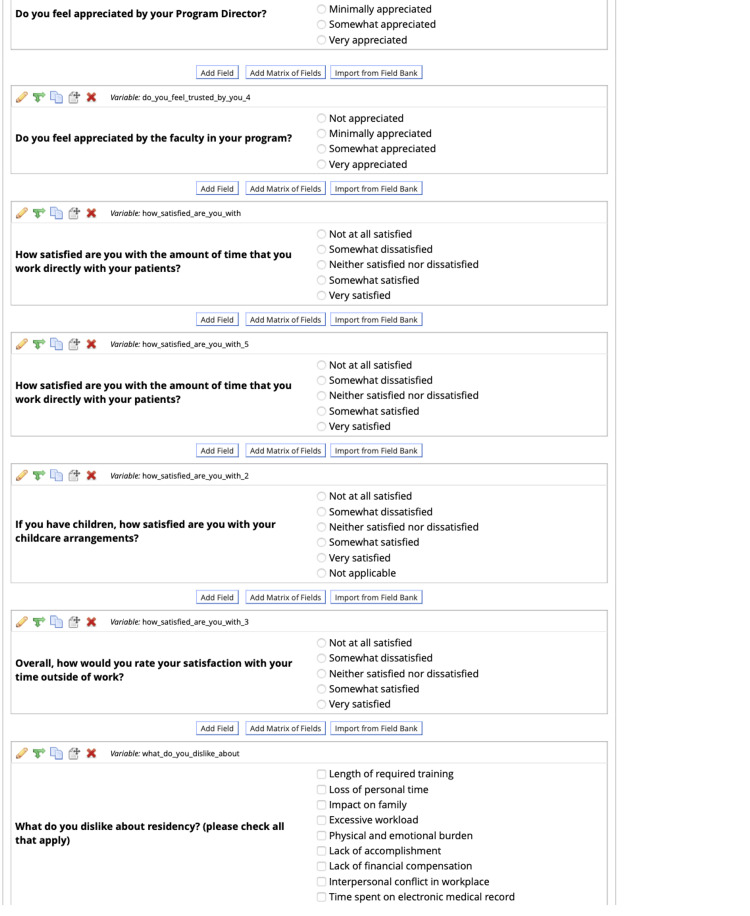
Survey Page 2

**Figure 5:**
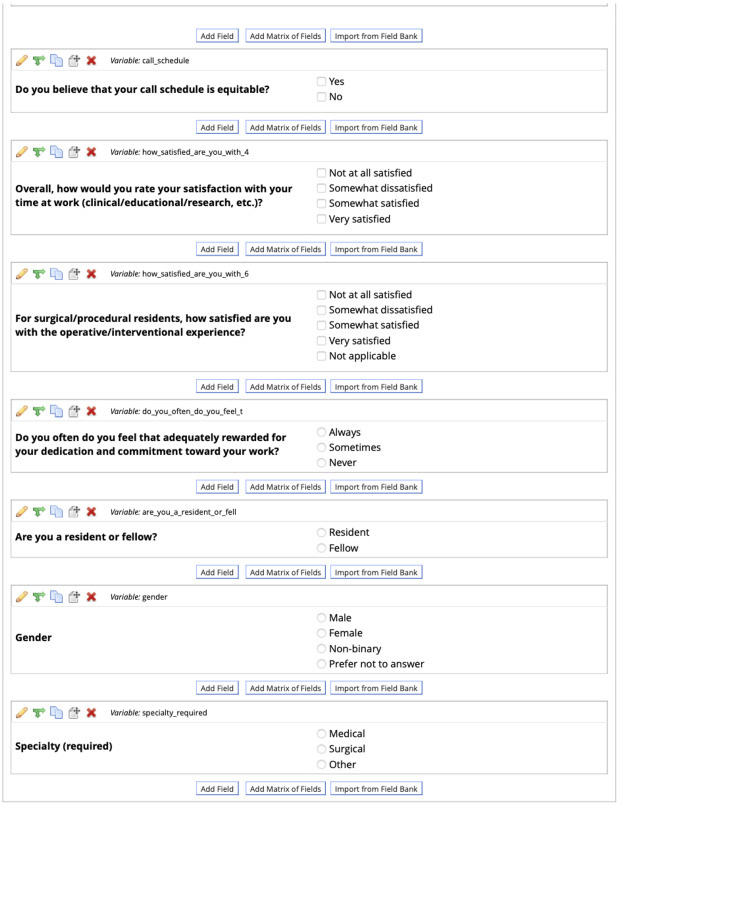
Survey Page 3
